# COVID-19 mortality surveillance in Lebanon

**DOI:** 10.1038/s41598-022-18715-6

**Published:** 2022-08-27

**Authors:** Linda Abou-Abbas, Zeina Nasser, Mario Baaklini, Lina Cheaito, Jeanette Karout, Hawraa Sweidan, Abbas Jouni, Nada Ghosn, Hamad Hassan

**Affiliations:** Epidemiological Surveillance Program, Ministry of Public Health, Beirut, Lebanon

**Keywords:** Respiratory tract diseases, Microbiology

## Abstract

Since the beginning of the COVID-19 pandemic, the Epidemiological surveillance program of the Lebanese Ministry of Public Health has launched a rapid surveillance system for collecting COVID-19-related mortality data. In this study, we document the Lebanese experience of COVID-19 mortality surveillance and provide an analysis of the epidemiological characteristics of confirmed deaths. The implementation of the rapid COVID-19 mortality surveillance system, data sources, and data collection were described. A retrospective descriptive analysis of the epidemiological characteristics of confirmed cases occurring in Lebanon between February 20, 2020, and September 15, 2021, was performed. Epidemiological curves of Covid-19 confirmed cases and deaths as well as the geographic distribution map of mortality rates were generated. Between February 21, 2020, and September 15, 2021, a total of 8163 COVID-19-related deaths were reported with a predominance of males (60.4%). More than 60% were aged 70 years or above. Of all deaths, 84% occurred at hospitals and 16% at home. The overall cumulative mortality rate was 119.6 per 100,000. The overall case fatality ratio (CRF) was 1.3%. Of the total deaths, 82.2% had at least one underlying medical condition. The top reported COVID-19 comorbidities associated with COVID-19-related deaths are cardiovascular diseases including hypertension (59.1%), diabetes (37.2%), kidney diseases including dialysis (11%), cancer (6.7%), and lung diseases (6.3%). The CFR was 30.9% for kidney diseases, 20.2% for cancer, 20.2% for lung diseases, 18.1% for liver diseases, 14% for diabetes, and 12.2% for cardiovascular diseases. Considering the limited human and financial resources in Lebanon due to the economic and political crisis, the rapid mortality surveillance system can be considered successful. Improving this system is important and would contribute to better detection of deaths from emerging and re-emerging diseases during health crises.

## Introduction

Coronavirus Disease 19 (COVID-19) is still an ongoing threat^1^. This transmissible infectious disease has devastated many countries and overwhelmed healthcare systems worldwide. Despite significant advancements in clinical research that have improved understanding and the management of COVID-19, controlling the ongoing spread of this virus and its variants has become a matter of increasing concern^2^.

During an epidemic, daily deaths count provides an important key indicator of the overall disease burden and tracking trajectory^3,4^. Thus, it is critical to establish a rapid mortality surveillance that can provide decision-makers with “insights into the full magnitude of the health consequences of an epidemic, and into disparities in disease burden across geographic and demographic groups^3^”. Rapid surveillance system can also help target, prioritize, and monitor the effectiveness of prevention and control strategies^4^.

In Lebanon, the first confirmed COVID-19 case was reported on February 21, 2020^5^, and the first confirmed death on March 10, 2020^6^. To date, September 15, 2021, a total of 614,069 confirmed cases have been reported and 8163 have died^7^.

At the beginning of the COVID-19 pandemic, the Epidemiological Surveillance Program (ESU) of the Lebanese Ministry of Public Health (MOPH) enhanced coordination for optimal COVID-19 mortality surveillance in order to issue daily timely data related to the pandemic. Thus, a rapid COVID-19 mortality surveillance system was implemented to collect data and inform decision-makers about the direction of the epidemic. In this paper, we aim to document the Lebanese experience of COVID-19 mortality surveillance system and provide analysis of the epidemiological characteristics of death cases from March 10, 2020 to September 15, 2021.

## Methods

During October 2021, a retrospective descriptive analysis related to COVID-19 mortality surveillance data was conducted. In compliance with relevant law on reporting of Communicable Diseases (law of 1957) and national legislation issued by the MOPH, no ethical approval is required as the data analysis falls under public health surveillance. This report conforms to the Strengthening the Reporting of Observational Studies in Epidemiology (STROBE) guideline.

### Implementation of the rapid mortality surveillance in Lebanon

The rapid mortality surveillance for COVID-19 relies on the extension of the routine communicable disease surveillance complemented by the inputs from the funerals services reporting, media and social media screening, and case investigation. The information was double-checked with the civil registration and vital statistics system, and the administrative services reporting system.

COVID-19 is considered as disease requiring immediate notification (within 24 h) to the ESU, including death event. The case definition for confirmed and probable COVID-19 cases is adopted from the world health organization (WHO) guideline^8^. The case definition for COVID-19 related death is: “a death resulting from a clinically compatible illness, in a probable or confirmed COVID-19 case, unless there is a clear alternative cause of death that cannot be related to COVID–19 disease (e.g. trauma)”^9^.

In each hospital, whether public or private, a focal person for communicable diseases reporting is appointed in charge of direct communication with ESU teams (central, provincial, and district levels). The same approach was adopted with the long stay healthcare facilities. Once a death is reported, investigation is initiated to check the conformity with the case definition and to collect additional information on the deaths and their contacts.

### Data sources

Indicator and event based surveillance were used for case finding. Indicator-based surveillance includes reports from healthcare facilities. Lebanese healthcare facilities from public and private sectors report to the MOPH on COVID-19 deaths via three channels: (1) The communicable disease surveillance system mentioned above; (2) The civil registration and vital statistics system, initiated since 2017, relying on weekly online anonymous death notification from hospitals, using the code U07.1 (from the International Classification of Diseases, Tenth Revision) for the diagnosis of COVID-19, virus identified^10^; (3) The administrative services reporting system, where hospitals report on daily basis bed occupancy and deaths using an online platform implemented in mid-2020 to monitor hospital readiness and capacity for COVID-19.

Event based surveillance has been used to supplement the indicator surveillance system to identify within the burials services or through media screening any death case related to COVID-19. The burial services were requested to promptly notify the ESU team about any COVID-19 related deaths. They were also requested to share with the ESU team a line list of all the available information regarding the death case including the name, date of birth, location or hospital’s name where the death occurred, date of death, and phone number. The ESU team was also asked to screen on a daily basis the following data sources: press releases, and social media feeds (i.e., Facebook, Twitter, Instagram) to search for death cases. In addition, deaths detected during COVID-19 case investigation were included. Death Cases of COVID-19 detected from the two unofficial sources (burial services, media screening) or during the investigation of confirmed cases were cross-checked and verified with the available recorded ESU data. If a new death case was detected, the ESU team was asked to initiate the investigation with the primary caregiver of the death case and collect all the required information.

### Data collection

Data collection on COVID-19 related deaths consists of two-stages: as first step, reporting form is filled to document any detected death (Annex 1). The form is filled by the healthcare facility focal person or by the ESU team (for death at home, death reported by the funeral services, or death collected from media screening). The collected data included: healthcare facility name, patient name, gender, birth date, place of residence, underlying conditions, and date of death. As second step, a detailed investigation is performed by the peripheral or central ESU team who contacted the healthcare facility and the relatives of the deceased to complete the corresponding information. A copy of the hospital discharge summary and the result of COVID-19 confirmatory test were requested. The investigation form included information on demographic characteristics, clinical presentation, underlying conditions, complications, hospitalization, exposure, vaccination status, and outcome. The vaccination status was stated: (a) “fully vaccinated” if the final dose of the COVID-19 vaccine was received at least 14 days from illness onset; (b) “partially vaccinated” if only first dose was received or if illness onset was between 14 days post 2nd dose; (c) “Not vaccinated” if no vaccine dose was received.

Data was entered into the District Health Information System of the MOPH, either at the source or by the investigation team. Data quality checks were performed routinely by the central ESU team to search for duplicates, aberrant, and missing values. Reports on COVID-19 mortality were published on a daily basis.

### Data analysis

COVID-19 confirmed cases and deaths occurring in Lebanon between February 21, 2020, and September 15, 2021 were extracted from the DHIS-2 of the MOPH. Epidemiological curves of Covid-19 confirmed cases and deaths were generated. The various phases of the epidemic and the control measures taken by Lebanese Government were presented. Daily average deaths were calculated for each phase. Age groups were defined as 0–9, 10–19, 20–29, 30–39, 40–48, 50–59, 60–69, 70–79, and ≥ 80 years. Frequencies, percentages, mortality rates and case fatality rates were used to report on the characteristics of deaths. Mortality rates were calculated per 100,000 population with the denominators corresponding to population estimates from the United Nations Population division in 2020. The data was analyzed using R version 4.0.2 (R foundation for Statistical Computing, https://www.r-project.org) and R Studio (https://www.rstudio.com/). QGIS version 3.10.13 (QGIS Development Team, http://qgis.osgeo.org) was used for mapping.

### Ethics approval and consent to participate

In compliance with relevant law on reporting of Communicable Diseases (law of 1957) and national legislation issued by the MOPH, no ethical approval is required as the data analysis falls under public health surveillance.

## Results

### Temporal distribution of COVID-19 confirmed and death cases

**Figure 1:**
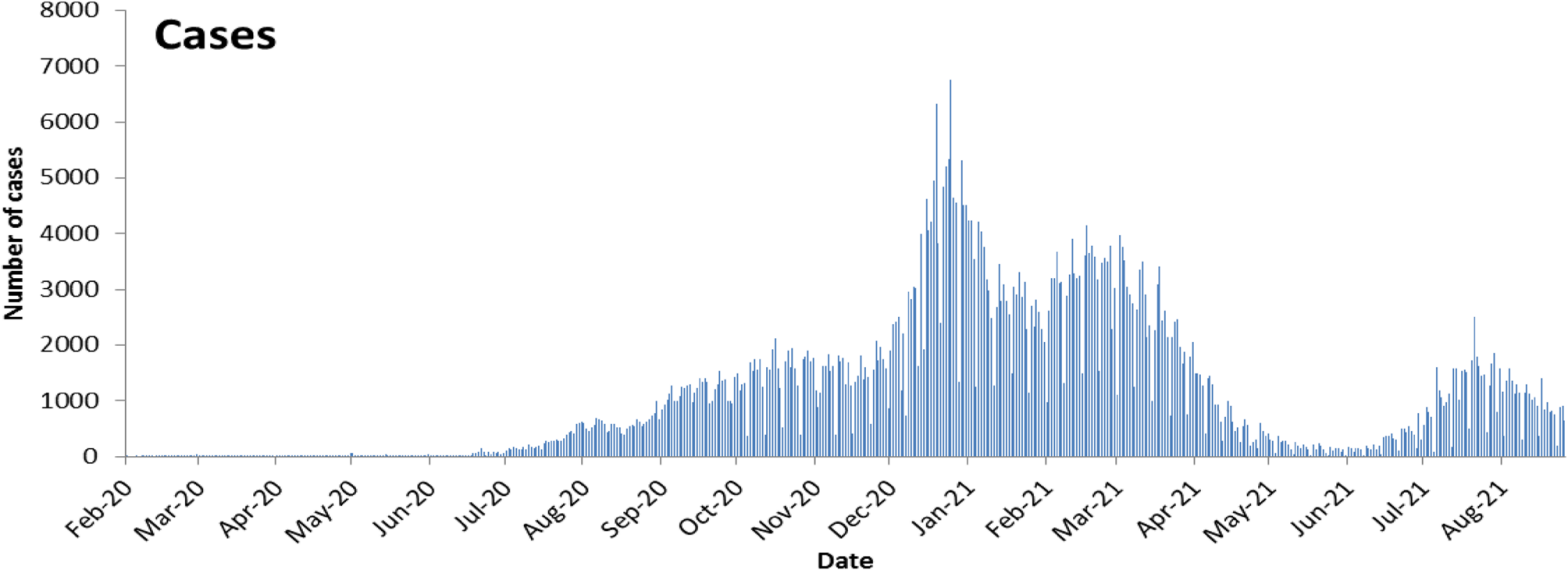
The epidemic curve of laboratory confirmed cases of COVID-19 in Lebanon starting from February 21, 2020 to September 15, 2021.

Between February 21, 2020 and September 15, 2021, a total of 614,069 confirmed COVID-19 cases and 8163 COVID-19 related deaths were reported to the ESU. Figure 1 illustrates the epidemic curve of laboratory confirmed cases in Lebanon starting February 21, 2020 and up to September 15, 2021.

**Figure 2:**
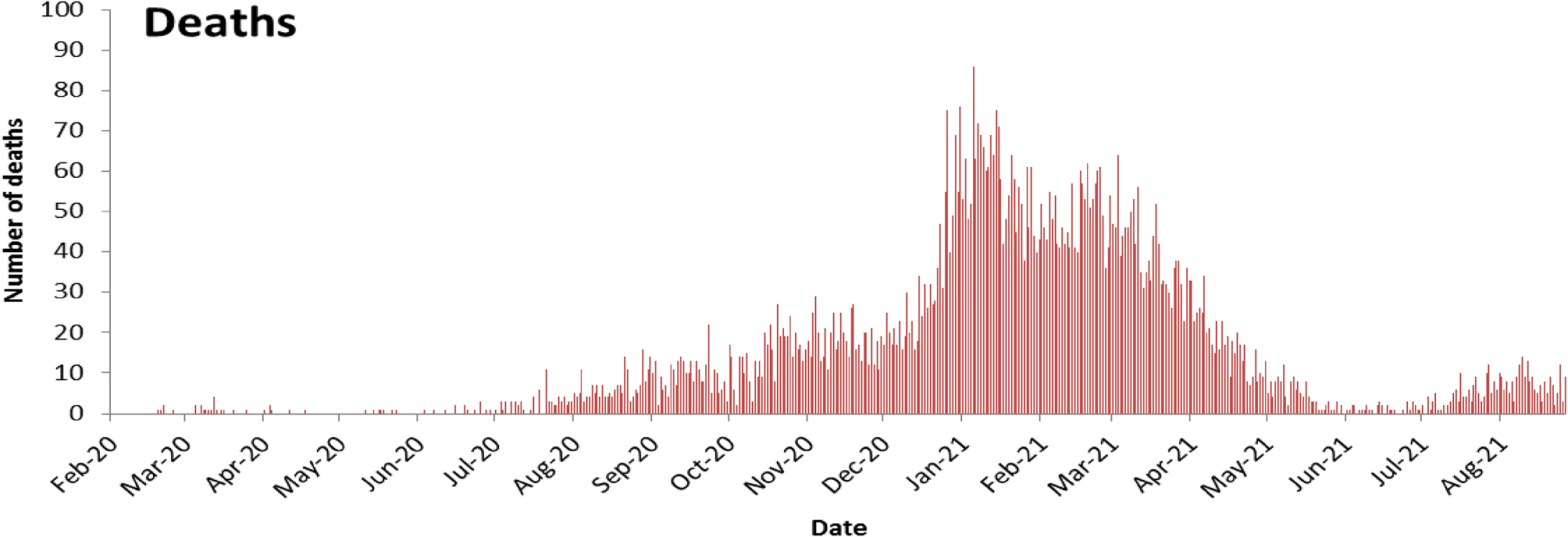
COVID-19 Death epidemic curve by day, Lebanon, February 21, 2020 to September 15, 2021.

The first death was reported on March 10, 2020. The death curve progressed in various phases (Fig. 2):

- Phase A from March to mid-July 2020: few deaths per day with a daily average of 0.3, ranging from 0 to 4, and representing 1% of all deaths. This phase reflects the introduction of the virus and detection of several clusters and the initiation of early lockdown measures. In the 21st February, the first COVID-19 case was confirmed among travelers. Early cases were isolated in designated hospital with contact tracing. On March 15, 2020, the emergency status was declared with school and business closure and travel restriction.
- Phase B from mid-July 2020 to end December 2020: with progressive increase of daily count, a daily average of 10.6 deaths, ranging from 0 to 30, and representing 22% of all deaths. This phase reflects the progression into community transmission following airport opening in July 2020. During that phase, the port of Beirut blast on August 4, 2020 contributed to the increase of the daily number of positive COVID-19 cases and later deaths. The explosion damaged many nearby hospitals, overcrowded healthcare facilities with patients.
- Phase C from January to mid-May 2021: corresponding to the big wave, with a daily average of 42.9, ranging from 10 to 98 (on February 4, 2021), and representing 70% of all deaths. This phase reflects the huge wave that has followed end 2020 social events, and implementation of the second general lockdown. The wave lasted from January to mid-May 2021.
- Phase D from mid-May to end July 2021: inter-wave period with a daily average of 3.7, ranging from 1 to 11, and representing 5% of all deaths.
- Phase E starting August 2021: new wave with a daily average of 6.8 deaths, ranging from 3 to 15, and representing 2% of all deaths.

### Distribution by age, gender and nationality

**Figure 3:**
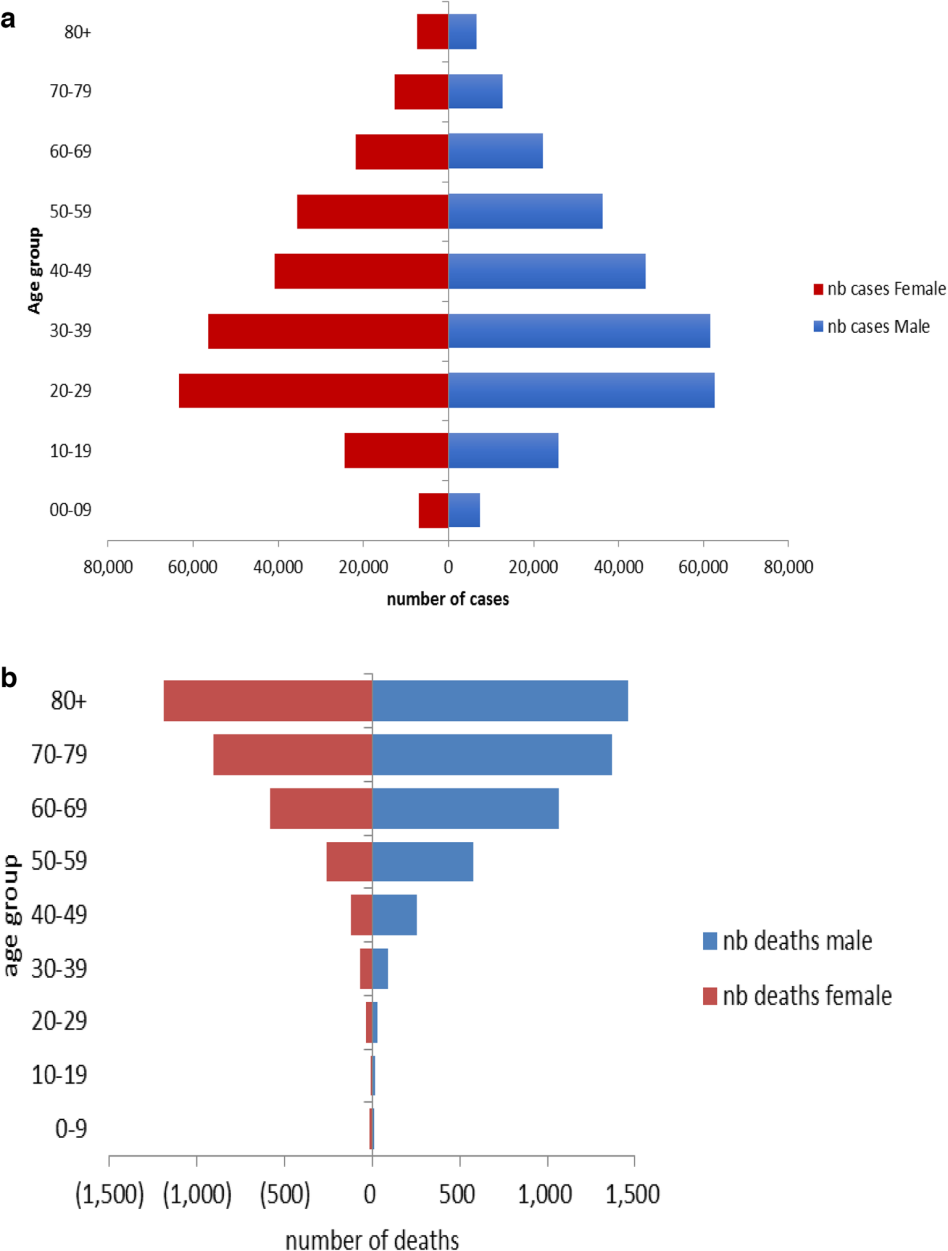
(**a**) Age-sex pyramid of confirmed COVID-19 cases (N = 614,069) reported in Lebanon between February 21, 2020 and September 15, 2021. (**b**) Age-sex pyramid of deaths (N = 8163) reported in Lebanon between March 10, 2020 and September 15, 2021.

Among the total 614,069 confirmed COVID-19 cases included in our study, 21.1% occurred in the ages between 20 and 29 years (Table 1). There were a higher proportion of male cases compared to females overall (1.06:1) and also within all age groups (Fig. 3a). The age pyramid of the deaths shows the predominance of older people and in particular among males (Fig. 3b). The distribution of deaths by age groups was as follows: 32.9% for 80 years and over, 28.2% for 70–79 years, 20.4% for 60–69 years, 10.4% for 50–59 years, 4.7% for 40–49 years, 2.0% for 30–39 years, 0.8% for 20–29 years, 0.3% for each 10–19 years and 0–9 years. The average age of the deaths was 72.4 years with a 95% Confidence Interval (CI) between 44 and 77. Regarding gender, 60.4% of deaths were males. The nationality distribution of the COVID-19 related deaths was as follows: 93.9% Lebanese, 2.9% Palestinian, 2.7% Syrian, and 0.2% Iraqi, 0.1% from Egyptian, 0.1% Bangladeshi, and 0.1% Ethiopian.

**Table 1:** Demographic characteristics of COVID-19 cases, deaths, mortality rate, and case fatality by age group and gender in Lebanon from February 21, 2020, 2020, till September 15, 2021.

	Cases	% of cases	Deaths	% of deaths (%)	Mortality rate/100,000	Case fatality rate % (%)
**All**	614,069		8163		119.60	1.33
**Age group**						
0–9	14,499	2.4	25	0.31	2.17	0.17
10–19	51,241	8.3	25	0.31	2.20	0.05
20–29	129,538	21.1	65	0.80	5.52	0.05
30–39	122,693	20.0	158	1.94	15.65	0.13
40–49	90,966	14.8	379	4.64	43.93	0.42
50–59	75,083	12.2	841	10.30	116.04	1.12
60–69	46,123	7.5	1646	20.16	367.60	3.57
70–79	26,596	4.3	2273	27.80	1074.33	8.55
80+	18,979	3.1	2656	32.54	2524.55	13.99
Unspecified age	38,351		95			
**Gender**						
Male	314,487	51.2	4931	60.41	143.52	1.57
Female	297,602	48.5	3231	39.59	95.32	1.09

Note: Not all cases have a reported age. Data corrections or updates can result in case records being removed and or updated from past reports and may result in subset totals differing from past publicly reported case counts. Case fatality rates are subject to bias as the accurate number of infected persons (denominator) is not available.

### Deaths by place: province of residence

**Figure 4:**
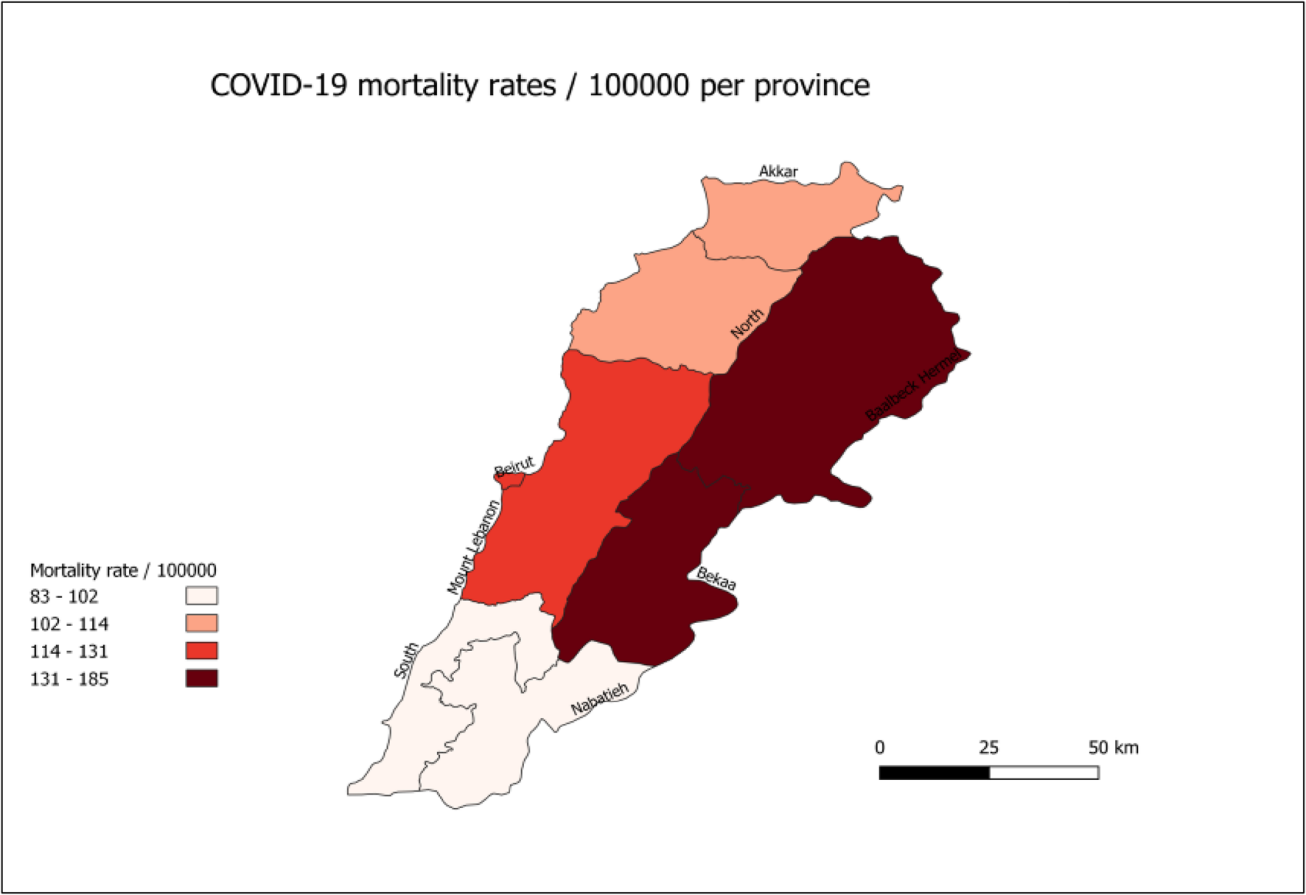
Cumulative mortality rate/100,000 population per province in Lebanon (March 10, 2020, to September 15, 2021).

Mortality rates showed variations across provinces. The highest cumulative mortality rate was reported in Bekaa (184.7/100,000), followed by Baalbeck-Hermel (136.8/100,000), Beirut (129.3/100,000), Mount Lebanon (121.2/100,000), North (106.6/100,000), Akkar (102.9/100,000), Nabatieh (100.2/100,000), and South (82.6/100,000) (Fig. 4).

### Mortality rates and case fatality ratios

The cumulative mortality rate was 119.6/100,000, 95% CI 117–122. Deaths related to COVID-19 varied considerably by age with the highest cumulative mortality rates among the elder population aged 80 years or above (2524.5/100,000), and the lowest one among children aged 0–9 years old (2.2/100,000). Regarding gender, the mortality rate was found to be higher among males (143.5/100,000) than females (95.3/100,000) (p-value < 0.0001) (Table 1).

The overall case fatality ratio (CFR) in the Lebanese population was found to be 1.3%. When data were stratified by age group, the CFRs appear similar for age groups 0 to 49 years, but ratios increased significantly starting age of 50 years and in particular for those aged 70–79 years old (8.5%) and those aged 80 years and above (14.0%) (p-value < 0.0001). Moreover, case fatality ratios differed by gender with a higher ratio for males compared to females (1.6% for males versus 1.1% for females (p-value < 0.0001) (Table 1).

### Comorbidities

Of the total deaths, 82.2% of the deaths had at least one comorbidity (Table 2). Cardiovascular disease was the most commonly reported comorbidity among confirmed COVID-19 cases both overall (59.1%) and as a single comorbidity (28.3%) followed by diabetes (37.2%), kidney diseases including dialysis (11%), cancer (6.7%), and lung diseases (6.3%). Diabetes and Cancer were reported as single comorbidity among 6.1% and 3% of the deaths respectively. The overall CFR was 4.7% for patients with comorbidities. The CFR was 30.9% for kidney diseases, 20.2% for cancer, 20.2% for lung diseases, 18.1% for liver diseases, 14.0% for diabetes, 12.2% for cardiovascular diseases, 8.5% for hematological diseases, 8.2% for overweight, 5.1% for immunodeficiency, 3.4% for asthma, 2.4% for post-partum, and 1.1% for pregnancy (Table 2).

**Table 2:** COVID-19 cases and deaths by comorbidities and case management in Lebanon, from February 21, 2020, 2020 to September 15, 2021.

	nb cases with comorbidities	% of cases/all cases (%)	nb deaths with comorbidities	% deaths/all deaths (%)	% case fatality ratio (deaths/cases) (%)
**Conditions**					
Comorbidities	142,196	23.16	6706	82.15	4.72
Cardiovascular (including hyperension)	39,707	6.47	4826	59.12	12.15
Diabetes	21,669	3.53	3036	37.19	14.01
Asthma	4689	0.76	158	1.94	3.37
Kidney diseases (including dialysis)	2902	0.47	897	10.99	30.91
Cancer	2717	0.44	550	6.74	20.24
Lung diseases	2566	0.42	518	6.35	20.19
Overweight	1556	0.25	127	1.56	8.16
Hematological diseases	861	0.14	73	0.89	8.48
Liver diseases	509	0.08	92	1.13	18.07
Immune deficiency	253	0.04	13	0.16	5.14
Pregnancy	1797	0.29	20	0.25	1.11
Post-partum	210	0.03	5	0.06	2.38
**Case management**					
Admission to hospital	31,482	5.13	7135	87.41	22.66
Admission to ICU	3562	0.58	1963	24.05	55.11
Mechanical ventilation	1495	0.24	1021	12.51	68.29

Note: cases with multiple comorbidities are counted for each condition.

### Case management

As of September 15, 2021, among the 8163 deaths, 7135 (87.4%) were admitted to hospitals, 1963 (24.0%) admitted to intensive care unit (ICU), and 1021 (12.5%) required mechanical ventilation. Of all deaths, 84% occurred at healthcare facilities and 16% at home. The CFR was 22.7% for COVID-19 patients who were admitted to hospitals, 55.1% in case of ICU admission, and 68.3% in case of mechanical ventilation (Table 2).

### Vaccination status

The MOPH launched the national vaccination campaign against COVID-19 on February 15, 2021. Of the total 3905 COVID-19 related deaths that occurred between February 15, 2021 and September 15, 2021, 1.5% of the death cases were fully vaccinated and 0.8% of the death cases were partially vaccinated.

## Discussion

The present study is the first to document the Lebanese experience of COVID-19 mortality surveillance and provide analysis of the epidemiological characteristics of death cases from February 21, 2020 to September 15, 2021. The extension of the national communicable disease surveillance system to COVID-19 related deaths and the rapid implementation of both indicator and event based surveillance of COVID-19 deaths were successful. Mortality data served as an indicator to inform policymakers on the epidemiological situation and to guide the processes of selecting and implementing the appropriate control measures. However, several challenges were encountered such the need of close coordination between various reporting systems, the burden of the COVID-19 on the burning out of the hospital staff including the turn-over, the economic crisis and the consequences on the shortage in power and internet supply.

Up to September 15, 2021, the death curve progressed in various phases: the phase A was characterized by identification of 3 major lineages: B.1 (20A clade) as predominant, B.4 lineage (19A clade) and B.1.1 lineage (20B clade)^10,11^. The phase C has witnessed the introduction and dissemination of the alpha variant^10,11^. The phase E was characterized by the introduction of the delta variant and progression into community transmission^11^.

Analysis of the collected data revealed that the COVID-19 mortality rate was higher in older adults, particularly men. Studies conducted by Abdel Ghaffar et al. in Egypt^12^ and by Al-Mudhaffer et al. in Iraq^13^ showed that male gender was associated with higher risk of mortality compared to female. They also found that older age and comorbidities were significant predictors of mortality among COVID-19 patients. In addition, other studies have documented that COVID-19-related death is higher in males than females and in older age groups, and the mechanisms behind these disparities have been postulated^14,15^. Another major characteristic that has been linked to poorer outcomes after COVID-19 infection is comrbidities^16–19^. Similar findings were reported by the US Centers for Disease Control and Prevention (CDC) which revealed that 94% of ICU deaths occurred among patients with one or more underlying health conditions^20^. Our findings are also in line with prior Italian^21,22^ and Chinese^23,24^ studies, implying that interventions to safeguard individuals with pre-existing medical disorders should be implemented to reduce COVID-19-related deaths.

Discrepancy in mortality rate was clearly shown across provinces with the highest cumulative mortality rate reported in Bekaa, followed by Baalbeck-Hermel. Discrepancy could be explained by the variation in the community spread of the virus and the applied preventive measures in each province. A nationwide population-based serosurvey study, conducted in Lebanon between December 7, 2020, and January 15, 2021, showed significant differences of SARSCoV-2 cases across provinces and the highest percentages of infected cases were found in Baalbek El-Hermel (34.1%) followed by Bekaa (21.0%)^25^. Therefore, the highest rate mortality could be explained by the increased number of infected cases in these provinces.

The overall CFR for COVID-19 in Lebanon was 1.3% and increased to 8.5% for the age group 70–79 and to 14% for the age group 80 years or older. A significant increase in the CFR was observed for individuals with comorbidities in particular for patients with kidney diseases, cancer, lung diseases, liver diseases, diabetes, and cardiovascular diseases. Calculating the CFR is challenging since the accurate number of infected persons (denominator) is not available. Therefore, measuring excess mortality^26^, and comparing the deaths figures with the previous pre-pandemic years can provide more comprehensive measure of the total impact of the pandemic on deaths. Moreover, it is necessary to conduct a survival analysis and to evaluate the impact of other extrinsic factors on COVID-19 deaths in this area to optimize our understanding^27,28^.

Genomic surveillance to combat COVID-19 is essential to detect and identify emerging variants and to provide actionable data for monitoring severe acute respiratory syndrome coronavirus 2 (SARS-CoV-2) community transmissions^29^. Thus, SARS-CoV2 genomic surveillance was undertaken with support from WHO involving public and private sectors and WHO-collaborating centers.

Continuous dissemination of the COVID-19 mortality data is crucial during this pandemic. Linking the death epidemic curve to the implemented social and public health measures provide insight in understanding the progression of the outbreak^30^. However, data quality is an important issue to be considered by the health policymakers. Moreover, comparison of data generated from each implemented surveillance system at different stages of the pandemic over time is important. Indicators should be defined for calculating the capacity, validity, and reliability of the COVID-19 surveillance system. Moreover, sensitivity, specificity and positive and negative predictive value are important measures recommended to evaluate the surveillance system. Lastly, it is important to educate healthcare workers on surveillance and proper death certificate issuance protocols to obtain accurate statistics, especially for COVID-19 patients with comorbidities.

## Conclusion

Countries need real-time awareness of the distribution and magnitude of the COVID-19 pandemic. Considering the limited human and financial resources in Lebanon due to the economic and political situation, our rapid COVID-19 mortality surveillance system can be considered as a success. Timely mortality surveillance is vital and would contribute to better detection of deaths from emerging and re-emerging diseases during health crisis. Our findings highlight the need for rapid and coordinated action during major outbreaks of infectious diseases to suppress and eliminate transmission and therefore minimize the detrimental effects on human health as well as social and economic activities.

## Supplementary Information

Supplementary Information.

## Data Availability

The data supporting study findings are the responsibility of the Epidemiological Surveillance Program (ESU) at the Lebanese Ministry of Public Health. Thus, restrictions apply to the availability of these data, which were used under license for the current study and are not publicly available. Data are, however, available from the authors upon reasonable request and with permission of ESU.

## Abbreviations

COVID-19: Coronavirus disease 2019
CRF: Case fatality ratio
ESU: Epidemiological Surveillance Program
MOPH: Ministry of Public Health
CI: Confidence Interval
ICU: Intensive Care Unit
SARS-COV-2: Severe acute respiratory syndrome coronavirus 2
