# Supplementary material for: COVID-19 mortality surveillance in Lebanon
Source: Sci Rep. 2022 Aug 27;12:14639. doi: 10.1038/s41598-022-18715-6 (PMC9419139; doi:10.1038/s41598-022-18715-6)
Supplement: Supplementary file 1 — Supplementary Information. [file 41598_2022_18715_MOESM1_ESM.pdf]

REPUBLIC OF LEBANON
MINISTRY OF PUBLIC HEALTH
EPIDEMIOLOGICAL SURVEILLANCE PROGRAM

ID number |__________|

COVID-19 Investigation Form

Republic of Lebanon – Ministry of Public Health

A. Reporter

Date of reporting: |____|____|__________| Facility name:

B. Patient information

First Name: Residence: □ Resident □ Visitor □ Refugee
Father Name: Caza of residence:
Family Name: Locality:
Date of Birth: |____|____|_________| Neighborhood:
Gender: □ M □ F Street name:
Nationality: Phone number:

C. Reason

Reason for testing: Nb of testing:

D. Signs and symptoms: □Yes □No

Onset Date |___|____|___________| □ Cough □ Dyspnea □ Sneezing □ Sore throat □ Abdominal pain
□ Fever (≥38°C) □ Rash □ Anosmia □ Diarrhea □ Headache □Arthralgia/Myalgia □ If other, specify:

E. Medical Care: □Yes □No

□ Medical consultation? Date |___|___|_________| □ Private □ ER □ Medical Center
□ Hospitalized? Since |___|___|_________| To |___|___|_________| Hospital Name:
□ Patient admitted to ICU? Since |___|___|_________| To |___|___|_________|
□ Mechanical ventilation? Since |___|___|_________| To |___|___|_________|

F. Complications: □Yes □No

□ Pathologic chest X-ray/CT □ ARDS □ Acute Renal Failure □ Multi-organ failure
□ Diagnosis of pneumonia □ Hypotension □ Cardiac arrest □ Other, specify

G. Risk factors/Exposure in the 14 days prior to illness onset: □Yes □No

□ Travel Where: Date back |___|___|________|
□ Contact with confirmed Covid19 case Who/Phone:
□ Contact with Severe Acute Respiratory Case Who:
□ Health Care worker Profession/Where:
□ Visited health care facility Where/Why:
□ Social event Specify:
□ Institutional outbreak Institution:

H. Underlying conditions: □Yes □No

□ Cancer □ Chronic liver dis. □ Heart disease □ Asthma □ Pregnancy □Regular smoker □ Overweight □ Other,
□ Diabetes □ Chronic lung dis. □ Hypertension □ Hematology □ Post-partum<6 wk □ Kidney Failure □Deficient immune specify:

I. Specimens and lab result

Date collection |____|____|_________| Specimen: Field testing: □Yes □No
Date confirmation |____|____|_________| Test: Laboratory:

J. Home Isolation

□ Home isolation? Since |____|____|________| To |____|____|______|
□ Institution isolation? Since |____|____|________| To |____|____|______| Place:

K. Profession - Active: □Yes □No

Occupation: Institution: Sector: Cluster: □Yes □No

L. Fatal outcome

□ Death, Death date |___|___|______| Place: Cause:

M. Close contacts

Name	Age	Relation	Locality	Phone	PCR test

I. Investigator Name and Date:

CS CamScanner
